# SpotitPy: a semi-automated tool for object-based co-localization of fluorescent labels in microscopy images

**DOI:** 10.1186/s12859-022-04988-1

**Published:** 2022-10-21

**Authors:** Alexia Akalestou-Clocher, Vivian Kalamara, Pantelis Topalis, George A. Garinis

**Affiliations:** 1 Department of Biology, University of Crete, Vassilika Vouton, 71409 Heraklion, Crete Greece; 2 Institute of Molecular Biology and Biotechnology, Foundation for Research and Technology-Hellas, 70013 Heraklion, Crete Greece

**Keywords:** Fluorescent microscopy, Co-localization, Image analysis, Quantification

## Abstract

**Background:**

In fluorescence microscopy, co-localization refers to the spatial overlap between different fluorescent labels in cells. The degree of overlap between two or more channels in a microscope may reveal a physical interaction or topological functional interconnection between molecules. Recent advances in the imaging field require the development of specialized computational analysis software for the unbiased assessment of fluorescently labelled microscopy images.

**Results:**

Here we present SpotitPy, a semi-automated image analysis tool for 2D object-based co-localization. SpotitPy allows the user to select fluorescent labels and perform a semi-automated and robust segmentation of the region of interest in distinct cell types. The workflow integrates advanced pre-processing manipulations for de-noising and in-depth semi-automated quantification of the co-localized fluorescent labels in two different channels. We validated SpotitPy by quantitatively assessing the presence of cytoplasmic ribonucleoprotein granules, e.g. *processing (P) bodies,* under conditions that challenge mRNA translation, thus highlighting SpotitPy benefits for semi-automatic, accurate analysis of large image datasets in eukaryotic cells. SpotitPy comes in a command line interface or a simple graphical user interphase and can be used as a standalone application.

**Conclusions:**

Overall, we present a novel and user-friendly tool that performs a semi-automated image analysis for 2D object-based co-localization. SpotitPy can provide reproducible and robust quantifications for large datasets within a limited timeframe. The software is open-source and can be found in the GitHub project repository: (https://github.com/alexiaales/SpotitPy).

**Supplementary Information:**

The online version contains supplementary material available at 10.1186/s12859-022-04988-1.

## Background

In fluorescent microscopy, fluorescent substances are examined in a microscope to visualize the dynamics of tissue, cells, organelles, and macromolecular assemblies [1]. In co-localization studies, the differential fluorescent labelling of molecules indicates their spatial proximity in the cell [2]. It is widely used to visualize the interplay between proteins, nucleic acids or lipids and elucidate their inferred topology, concentration or regulatory function. A number of analysis and quantification methods exist that process and analyse the spatial distribution of different biomarkers within cells [3–6].

Co-localization analysis typically exploits pixel- or object-based approaches to assess the extent to which different fluorescent labels overlap [7, 8]. Pixel-based algorithms calculate the correlation coefficients e.g., Pearson, Manders coefficient, Costes, Van Steensel or Lis’ approach by pixel-to-pixel comparison of the channel's intensity values [9–12]. In spite of their widespread use and relative ease of operation, correlation coefficients are limited to global estimates on the degree to which co-localization occurs and do not allow for spatial exploration. On the other hand, object-based co-localization analysis (OBCA) has the ability to define and quantify the degree of co-localization between individual objects in a statistically robust manner [7, 13, 14].

OBCA algorithms however, are more technically demanding [15]. With that being said, one of the main perquisites, of OBCA algorithms is a-priori punctual cell segmentation step. Depending on the purpose of the analysis, software implement different segmentation methods which can vary from manual or pixel-based [5, 14, 16, 17] to more automated approaches [18–20]. Besides the flexibility manual labelling provides, semi-automated versions grant an increased reproducibility, robustness and scalability. Through the use of a semi- automated approach, SpotitPy has the potential to analyse larger datasets in a limited timescale hence rendering it scalable.

Here we developed SpotitPy, a novel OBCA software that can assess the exact number of identified co-localized objects in two dimensions. The software implements novel semi-automatic segmentation methods, which allow accurate cell body segmentation. Since manual segmentation is not required, reproducibility and scalability can be achieved. Coupled with novel de-noising techniques, SpotitPy can assess an unbiased, high-throughput analysis of co-localized objects in large image datasets. The tool is freely available and operates in a user-friendly graphical user interface (GUI) or a similar command line interface (CLI) version that allows for higher flexibility in parameter selection.

## Implementation

### SpotitPy development

The code for SpotitPy was written in Python release 3.8 [21] and compiled in Spyder 4.2 [22], a workplace for writing and compiling code in Python. SpotitPy was developed with open-source packages that are available for Python and are all imported in the beginning of the script. These are NumPy, pandas, matplotlib, Trackpy, Cellpose and Wavelet-Based Background Noise Subtraction (WBNS) [18, 23–26]. Tkinter was used to generate the code for the object-oriented interface. The computational time required is mainly defined by the segmentation step and varies greatly based on the depicted cell size and population.

### Mice, primary cell cultures and sample preparation

All animal studies were approved by an independent Animal Ethical Committee at the Foundation for Research and Technology—Hellas (FORTH) and abided by the ARRIVE guidelines (Additional file 1). Animals (*Mus musculus*, strain C57BL/6, males and females) were bred in the IMBB-FORTH animal facility unit. The endpoint of the animal experiment is euthanasia by CO_2_ inhalation. Bone marrow delivered macrophages (BMDMs) were differentiated from bone marrow precursors. Bone marrow cells were isolated from mice femurs and tibias and cultured for 7 days in Dulbecco’s modified Eagle’s medium (Thermo fisher Scientific, Carlsbad, CA, USA) containing 10% fetal Bovine serum (Gibco, Thermo fisher Scientific, Carlsbad, CA, USA) and GMCSF to induce their differentiation into macrophages. On the seventh day, macrophage cultures were treated with rapamycin (2 μM) for 24 h (Tocris Bio-techne, Greece).

### Immunofluorescence staining

For immunofluorescence experiments, BMDMs, were fixed in 4% formaldehyde rendering the system non-dynamic while preserving it for imaging purposes. Subsequently, cells were permeabilized with 0.5% Triton-X and blocked with 1% BSA. After overnight incubation with primary antibodies, secondary fluorescent antibodies were added and DAPI was used for nuclear counterstaining. The antibody against DDX6 (14,632-1-AP, IF:1/300) was from Proteintech, USA. The antibody against DCP1A (H00055802-M06, IF:1/300) was from Novus, USA. The goat anti-rabbit IgG Alexa Fluor 488 (A-11001; IF: 1/1000) and goat anti-mouse IgG Alexa Fluor 555 (A-21422; IF: 1/1000) were from Invitrogen, USA.

### Image acquisition

Images of the stained cells were captured by using Laser Scanning Confocal Microscope unit (TCS SP8, Leica Microsystems, Germany) under oil immersion objective lenses allowing 63 × zoom. The gain of all detectors was adjusted in 100% and remained constant for all image acquisitions. The resolution for the image acquisition, was set at 1024 × 1024 pixels, so that 4–5 pixels represent 0.5μm^2^ of the object area when using 63 × objective lenses. The scanning speed was set at 600 Hz, the pinhole was adjusted to 1 Airy unit and the z-step size to 0.5 nm/slide. The image colour depth was 8 bits and the scanning was performed bi-directionally. The fluorophores used in staining (DAPI, Alexa-448, Alexa-555), have excitation wavelengths at 405, 488 and 561. To avoid the compensation bias due to overlapping emission range, sequential scanning was preferred. The number of z-stacks taken for each image were in the range of 8–25 depending on the cell size, to ensure the maximum depth resolution in each cell.

### Testing of SpotitPy

SpotitPy was developed and validated using confocal images of BMDMs to identify the presence of P-bodies under challenging conditions. Unpublished images of BMDMs were used to illustrate the following computational cascade as well as the software parameters and the validation. Additionally, a comparative analysis was performed with some of the already existing available co-localization tools as well as a manual analysis for validation purposes. Images portrayed in the respective figures have amplified intensities for optimal depiction.

## Results

### Overview of SpotitPy workflow inputs

**Fig. 1:**
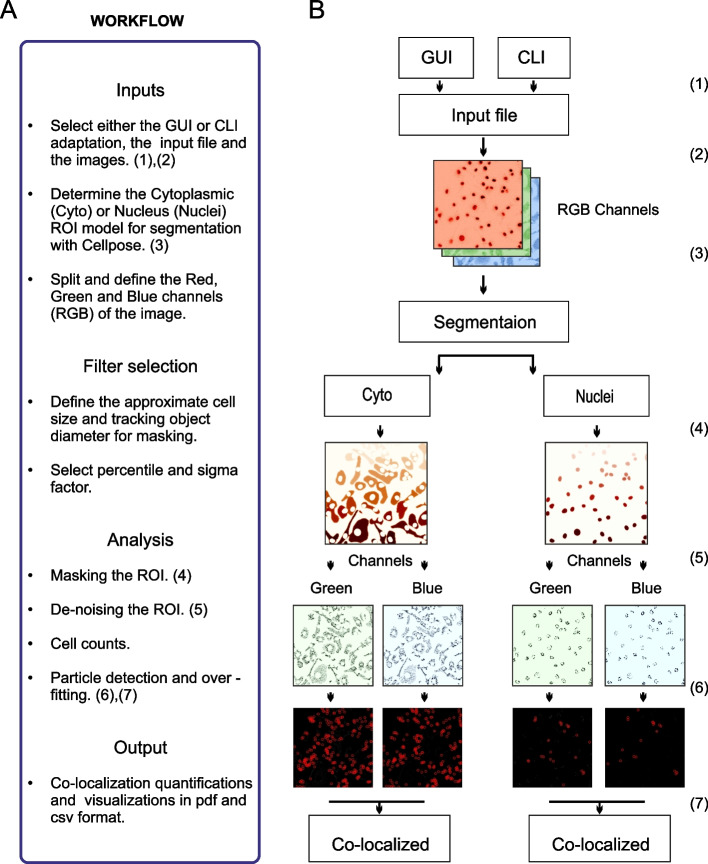
Overview of the SpotitPy software design in Python. **A** Workflow description of SpotitPy. After selecting either the GUI or CLI adaptation of the software (1), the user has to provide the input images, which meet the required perquisites (2). Parameters of the experimental setup have to be defined. These include selecting the model of segmentation (3) and defining the appropriate thresholds. Once completed, the software will initiate the analysis by identifying the cellular compartments. It will then create the mask for the ROI selection (4), de-noise the remaining image (5), estimate the number of cells and conclude with the particle tracking step in each channel. Finally, each channel’s detected particles (6) will be compared to identify the co-localized particles (7). All results can be found in the output generated data files. **B** Visual interpretation of each step with the corresponding numerical identifiers. Matplotlib was used for image visualization

As shown in the schematic representation of the tool-chain workflow (Fig. 1) two different adaptations of SpotitPy are available to select. Either: (1) a user-friendly graphical user interface (GUI) version (Additional file 2A) which was developed in order to facilitate all users, regardless of their computational skills, or (2) a similar command line interface (CLI) version that allows for higher flexibility in parameter selection (Additional file 4A). Detailed instructions for use and implementation of SpotitPy can be found in the GitHub project repository (https://github.com/alexiaales/SpotitPy).

### Input prerequisites

An input file is mandatory. The image files should meet the following requirements: (1) be in a Red, Green and Blue (RGB) format, (2) have no more than 30 compilations in each photograph taken at a set interval between the first and last planes of focus (z-stacks) and (3) be in a LIF format.

### Segmentation: determination of the region of interest

**Fig. 2:**
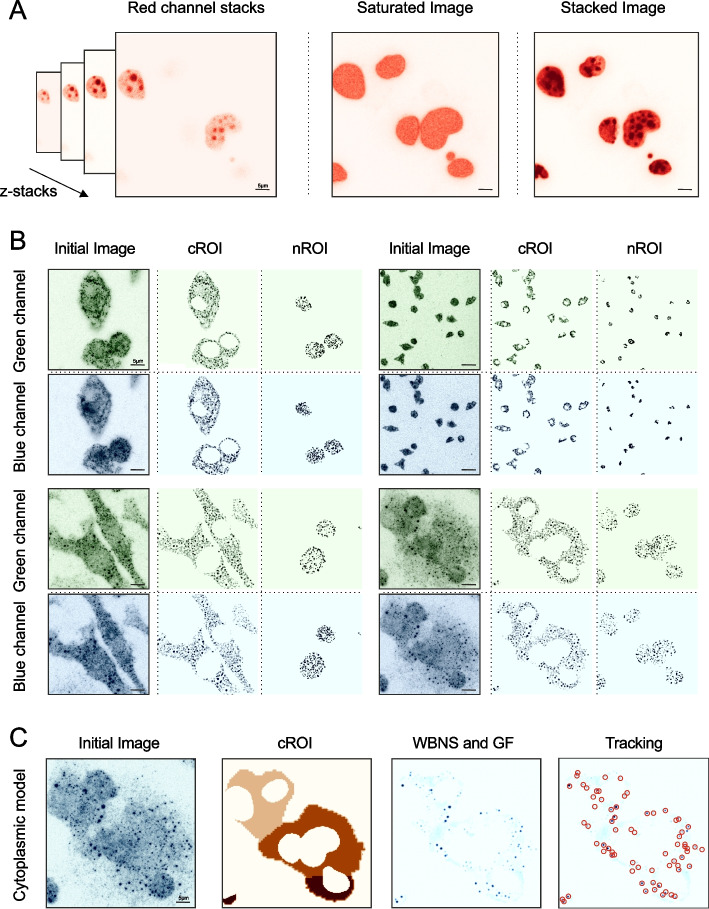
Segmentation for ROI selection and masking. **A** Comparison of the stacked and saturated image. Low signal regions are visually enhanced in the saturation step for more precise segmentation. **B** ROI selection example in both models (cROI: cytoplasmic ROI, nROI: nucleolar ROI). The initial image represents the input in each channel respectively (Green Channel and Blue Channel). **C** De-noising process before the tracking step. Once defining the cROI on the initial image and applying the mask, we further clear the image by applying a WBNS and the Gaussian filter which creates a more precise image. Lastly, we perform the tracking step. Matplotlib was used for image visualization

Animal studies and immunofluorescence experiments were performed as previously described [27–32]. Co-localization analysis often requires an initial image segmentation step to minimize unsolicited occurrence of false positives by incorrectly identifying fluorescent signals in irrelevant regions. To appropriately identify the regions of interest (ROI), a subspace of the complete image is selected by segmentation. Segmentation approaches vary from manual annotation to more automated ones, with deep learning methods outperforming traditional segmentation algorithms, such as the watershed-segmentation [33, 34]. To overcome the time-consuming and prone-to-error manual labelling of the ROI, we used Cellpose, a deep-learning algorithm that segments a wide range of cell types and subcellular compartments, to construct two different segmentation pipelines (Fig. 1) [18]. The first implemented segmentation procedure, which is referred to as “Cyto”, is used to identify objects that are only present in the cytoplasm. Likewise, the second segmentation pipeline, which can be selected by choosing the “nuclei” option, is used for object identification in the cell nucleus. Example segmentations for ROI selection with both pipelines are shown in Fig. 2B. Staining of cells with the appropriate cytoplasmic or membrane markers is required to correctly segment the cell body or the nucleus image volume. The segmentation step is applied on saturated rather than stacked images, where the intensity of each stack is added to the previous one and does not represent the local maxima (Fig. 2A). As a result, the saturation step provides images with higher overall intensities amplifying the stained regions, which is an essential step for accurate segmentation and masking (Fig. 2C).

### Selecting thresholds

**Fig. 3:**
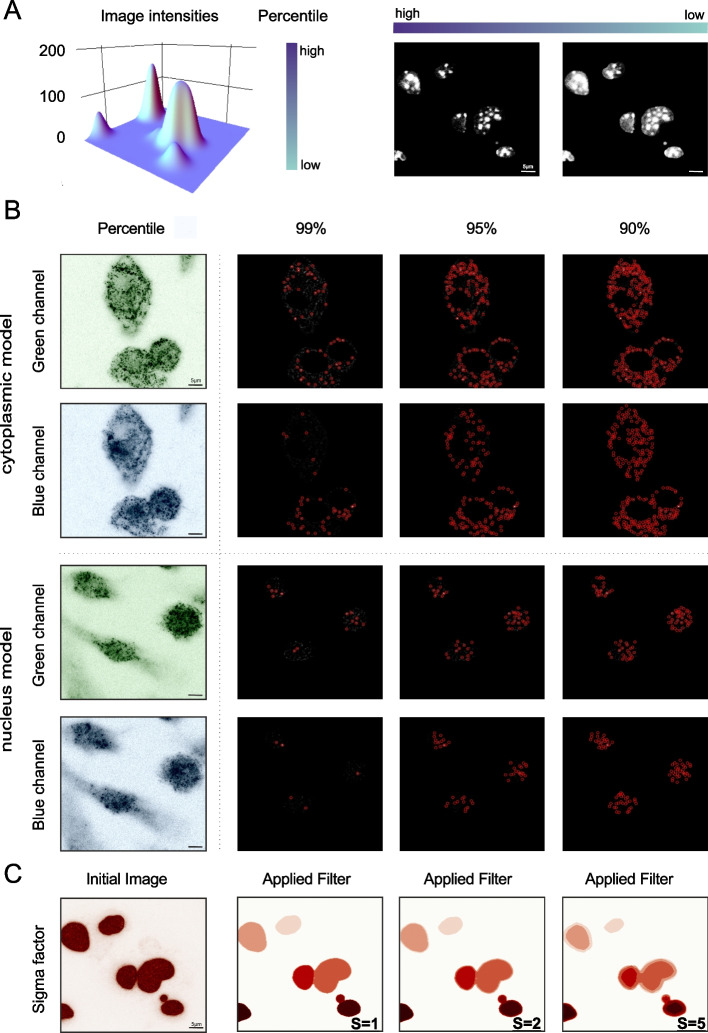
Differential thresholding on images. **A** 3D Intensities surface plot. The percentile bar indicates the intensities that are being cut off which are also interpreted in terms of the images’ output. In the first image only high intensities are included while the second image incorporates lower intensities. **B** Visual representation of a gradual percentile reduction in the tracking step. High percentile thresholds lead to solely high intensities depiction, which allows only a small number of particles detection. Similarly, lower percentile threshold allows more intensities to be included in the final image, which will unavoidably increase the number of identified particles in the tracking step. High percentiles are ideal for noisy images while lower ones might deal with images where the staining is not distinctive. **C** Visual representation of different sigma values of the Gaussian filter. As the sigma increases the edges are being smoothed out. Matplotlib was used for image visualization

Threshold selection is critical in co-localization analysis as small differences often lead to considerable downstream differentiations. Different thresholds are required depending on the staining method, the image quality or the type of experiment conducted to obtain biologically meaningful results. The percentile selection refers to the percentage of intensities that are being cut off (Fig. 3A). The higher the percentile, the fewer the intensities that will be selected and the particles that will be detected during the particle identification step (Fig. 3B). Elevated percentiles are required to avoid artefacts in noisy images, whereas lower ones are preferable in those cases where the staining is not vivid. To achieve further noise reduction as well as blurring or edge smoothing, we apply a Gaussian filter imported by scikit-image [35]. The Gaussian filter is applied after selection of the sigma factor, which stands for the noise variance. Higher selected sigma, performs more attenuation on high frequency signals (Fig. 3C). The user is advised to perform a visual inspection of the selected images before threshold selection.

### Pre-processing

Non-representative intensity variations are ubiquitously present due to the inherent characteristics of the procedure, from the Poisson distribution, which characterizes the incoming photons to the fluctuating experimental conditions. To reduce the intensity variations and amplify weak signals, we chose to incorporate the novel Wavelet-Based Background Noise Subtraction (WBNS) algorithm as a pre-processing step. Through WBNS we achieve noise reduction and enhancement of the visual appearance of fluorescent signals without deteriorating their content [36]. By combining cell body segmentation to define the ROI, Gaussian filtering and the WBNS as a set of pre-processing steps to the quantitative analysis, we succeed in generating relatively noise-free fluorescent images that subsequently are used for particle tracking (Fig. 2C).

### Molecule tracking and co-localization analysis

To analyse multi-fluorescent image data for co-localization purposes, the emitted signals in each channel need to undergo independent particle tracking. The detected particles' spatial locations are saved in the form of a Cartesian coordinate system and are assigned to a unique identification number. To quantify the degree to which different fluorescent labels overlap, we compare the particle tracking step in each separate channel with Trackpy Python library [26]. Two particles are characterized as co-localized when the two resulting data frames are compared and their coordinates do not exceed the specified maximum displacement, which is the equivalent to the defined particle's diameter. The CLI version allows further specification of the displacement range (Additional file 4A). Particle identification in independent channels follows a linear model (Additional file 3A). Their 2D co-localization follows a nonlinear pattern, reaching a plateau where the increase of fluorescent signal does not lead to a proportional increase in identified co-localized particles (Additional file 3B). The true number of co-localization, therefore, lies in the plateau region (Additional file 3C). The number of particles, which are identified in each channel as well as the number of particles qualified as co-localized, are available in the output csv data file (Additional file 4B). For visual inspection, a pdf data file displays the detected particles in each channel with red circular overlay.

### Statistical evaluation of cell number

Following segmentation and particle detection, the estimation of the depicted number of cells is required to perform a statistical approach of the mean co-localized objects per cell. Here, we propose an automated approach to extract the number of uniquely identified nuclei from the segmentation step. However, images consisting of many cells bear a high possibility of fractional cell representation. Some of the cells' area may not be included in the image, thus providing misleading quantitative values. To address this, instead of automatically removing these cells from our analysis, we incorporated the partially depicted ones as a fraction in comparison to the average nuclei size. This method led to an accurate estimate of the number of cells depicted in an image and was validated by visual inspection (Additional file 3D).

### Resulting outputs

Once the analysis is concluded a message appears on the screen letting the user know that the output files are available. Results consist of a csv data file with the statistical and quantitative information as well as a pdf data file for visual inspection of the performed analysis. In the command line version, the user can define the annotation of the output files. Images and quantifications generated by the program can be saved. The csv data table lists various fields as seen in Additional file 4B. To demonstrate the outputs, indicative files are provided in the created GitHub depository.

### Application of SpotitPy and validation

**Fig. 4:**
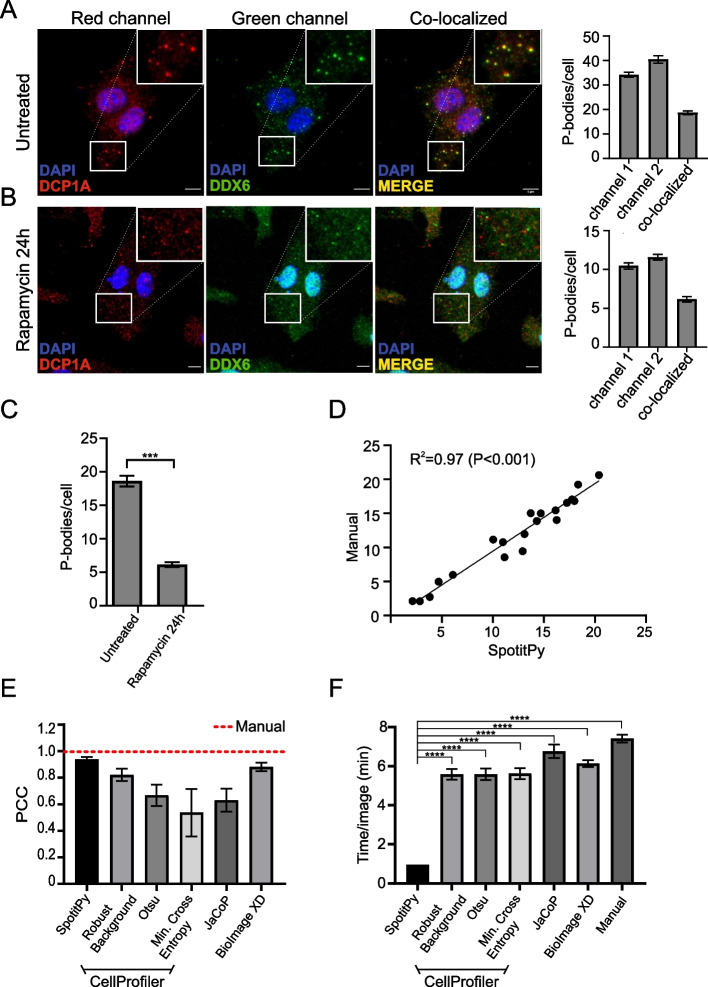
SpotitPy performance on confocal images for P-bodies identification under various conditions. **A** Confocal images of detected P bodies in untreated BMDMs. Detected particles in both channels are indicated respectively. P-bodies are depicted in the co-localized image where there is an overlap of the detected particles in both channels. The resulting yellow particles are defined as P-bodies. Inserts at the top right show a magnification of the boxed area of the field. Bar plot of the detected particles in each separate channel and the co-localized identified particles. **B** Confocal images of detected P bodies in rapamycin-treated BMDMs for 24 h. Detected particles in both channels are indicated respectively. P-bodies are depicted in the co-localized image where there is an overlap of the detected particles in both channels. The resulting yellow particles are defined as P-bodies. Inserts at the top right show a magnification of the boxed area of the field. Bar plot of the detected particles in each separate channel as well as of the co-localized identified particles. **C** Comparative bar plot of the total number of identified P-bodies in untreated and rapamycin-treated BMDMs (as indicated). **D** Scatterplot of co-localization detection by SpotitPy and manual counting in BMDMs (n = 20). Linear regression fit analysis with R^2^ = 0.97 (*P* < 0.001). **E** Comparison of SpotitPy to traditional analytical methods for object based co-localization analysis identifications. Pearson Correlation Coefficient (PCC) and squared standard error were estimated in a bar chart across methods (n = 9). **F** Average execution time estimation (in minutes) required to analyse an image (n = 9)

To test the accuracy and sensitivity of SpotitPy on fluorescent microscopy, we used the software to detect the presence of P-bodies in the cytoplasm of BMDMs under various conditions. Unlike other cytoplasmic entities, P-bodies are highly dynamic membrane-less aggregates consisting of ribonucleoproteins and mRNA that are often challenging to detect [37]. P-bodies are defined by the co-localization of DEAD-Box Helicase 6 (DDX6) found in P-bodies with the decapping protein 1A (DCP1A) involved in mRNA decay [38]. SpotitPy successfully identified the significant decrease in the number of P-bodies per cell when BMDMs were treated with rapamycin, a natural anti-fungal antibiotic known to inhibit mRNA translation and P-body formation. This decrease is also consistent with the existing literature [39, 40] (Fig. 4A–C). Using SpotitPy, we were able to accurately reproduce the results obtained when P-bodies were manually counted in the samples (Additional file 3E). This was further reflected in the significant positive correlation e.g. R^2^ = 0.97 (*P* < 0.001) between SpotitPy counting and manual counting using linear regression analysis (Fig. 4D). We additionally performed a comparative analysis of our results to traditional object based co-localization analysis methods by calculating the Pearson Correlation Coefficient (PCC) for all methods. SpotitPy was able to yield the most accurate results in comparison to the commonly used methods (Fig. 4E). The reported execution times while comparing SpotitPy to the available software highlighted the ability of SpotitPy to perform an analysis with in a limited timeframe (Fig. 4F).

Thus, SpotitPy performs a semi-automatic, accurate co-localization analysis on fluorescent microscopy images though precise ROI selection, efficient de-noising, cell counts and particle tracking.

## Discussion

Fluorescent microscopy is a rapidly evolving technology that requires the development of specialized software applications to capture, document and analyse coloured or monochrome images. Here, we developed SpotitPy, a free and open-source tool, which allows for a semi-automated, object-based co-localization analysis in fluorescent microscopy images.

SpotitPy performs a semi-automated object-based co-localization analysis that provides the absolute number of co-localized particles instead of relative correlation coefficient estimates, which are prone to subjective interpretations. The semi-automatic segmentation of the ROI allows the co-localization analysis of large datasets achieving high scalability and reproducibility; in manual segmentation, the latter is challenging as sample blindness often introduces fluctuations in quantification. By setting the size of nuclei and particles as well as defining the threshold of all images in the beginning, SpotitPy it suitable for larger studies where many biological images need to be analysed at once.

The software incorporates recently established algorithms that reduce the incoming signals from background and non-cell objects while enhancing the label’s intensities generating a relative noise-free image for more accurate and robust particle detection. Additionally, SpotitPy allows the estimation of the exact number of cells depicted in one image, hence automatically providing the user with the statistical information of the average co-localized particles per cell. To validate the performed analysis and/or re-evaluate the input parameters, the user is provided with a visual output of the analysis. SpotitPy accuracy was tested and successfully validated through a co-localization analysis for the detection of P-bodies. Unlike with membranous cytoplasmic structures, P-bodies are detectable by the cytoplasmic co-localization proteins making their detection challenging and prone to bias. Using SpotitPy, we were able to reproducibly quantify the absolute number of P-bodies per cell under various experimental conditions in a short time frame, highlighting its accuracy and sensitivity.

There are some limitations that the user needs to acknowledge when using this software. SpotitPy can measure the co-localization of two reporters in two separate channels. In case of more channels the software cannot asses the information. The tracking step is best suited for tracking spherical objects, so it is preferable that one avoids irregular particle shapes. SpotitPy is unable to distinguish objects with similar XY coordinates but different Z locations. Such objects will be merged in the stacking step and fail to be treated as separate entities. Finally, inappropriate segmentation leads to miscalculations highlighting the importance of providing images with low heterogeneity and sparsely distributed cells. SpotitPy’s pre-processing techniques can only partially ameliorate the quality of the input images. Thus low resolution, extremely noisy or non-representative images may lead to inaccurate results, regardless of the applied quantification tool.

## Conclusions

Taken together, SpotitPy is a valuable contribution to the publicly available repertoire of software for fluorescent microscopy co-localization analysis in either a GUI or CLI adaptation. SpotitPy is a novel user-friendly tool that performs a semi-automated, hence reproducible with robust quantifications, object-based co-localization analysis for large datasets within a limited timeframe. The software is open-source and is shared on GitHub to allow researchers to add new methods, features and ideas that will take this tool to new directions.

## Availability and requirements

- **Project name:** SpotitPy.
- **Project home page:** https://github.com/alexiaales/SpotitPy
- **Operating system(s):** Windows 10.0
- **Programming language:** Python 3.0
- **Other requirements:** NumPy, Pandas, Matplotlib, Trackpy, Cellpose and WBNS.
- **Any restrictions to use by non-academics:** licence needed.
- **License:** GNU GPL v3.

## Supplementary Information

**Additional file 1**. ARRIVE guidelines. Completed ARRIVE checklist and description.

**Additional file 2**. Graphical User Interface and segmentation. (A) An example of the graphical user interface (GUI). It consists of four separate and interactive panels, which can be used for parameter selection and visual inspection of the analysed images. (B) Segmentation examples of both the cROI and nROI in cells of various sizes. Matplotlib was used for image visualization.

**Additional file 3**. Enumeration of detected particles and cells. (A) Dot plot depicting the linearity of the percentile selection and particle tracking in both channels. (B) Dot plot depicting the nonlinearity of the percentile selection in the co-localized particles (C) Comparison of the nonlinear trend followed by the identified co-localized particles (red line) and the linear trend of the detected particles in the blue channel (blue line). (D) Comparative analysis of the total number of cells identified in each image by SpotitPy and by manual detection for validation; the dark and grey columns represent the results obtained by SpotitPy and average manual count (n=3), respectively. The images are available in the GitHub repository. (E) Comparative analysis of the total co-localized particle counts of P-bodies in BMDMs by SpotitPy as well as manual count; the dark and grey columns represent the results obtained by SpotitPy and manual count, respectively (n=9).

**Additional file 4**. Depiction of the software’s output and version’s parameters. (A) Comparison of the graphical user’s interface and command lines interface parameter settings. (B) SpotitPy excel output results. Indices explain each column.

## Data Availability

The source code with all the necessary instructions and comments are available at the following GitHub project repository: https://github.com/alexiaales/SpotitPy

## Abbreviations

ROI: Region of interest
OBCA: Object-based co-localization analysis
WBNS: Wavelet-based background noise
RGB: Red, green and blue
GUI: Graphical user interface
CLI: Command line interface
BMDMs: Bone marrow derived macrophages
PCC: Pearson correlation coefficient
