# Supplementary figures and images for: SpotitPy: a semi-automated tool for object-based co-localization of fluorescent labels in microscopy images
Source: BMC Bioinformatics. 2022 Oct 21;23:439. doi: 10.1186/s12859-022-04988-1 (PMC9587566; doi:10.1186/s12859-022-04988-1)

A

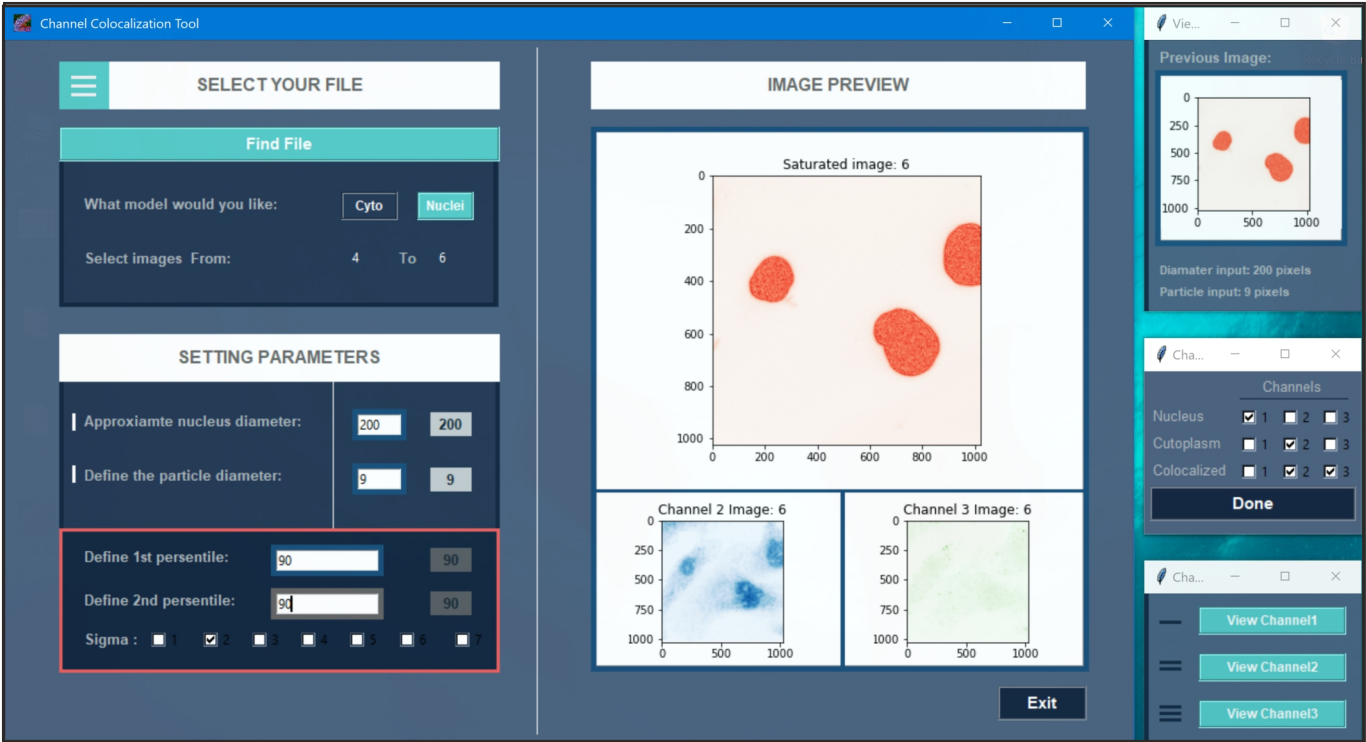

B

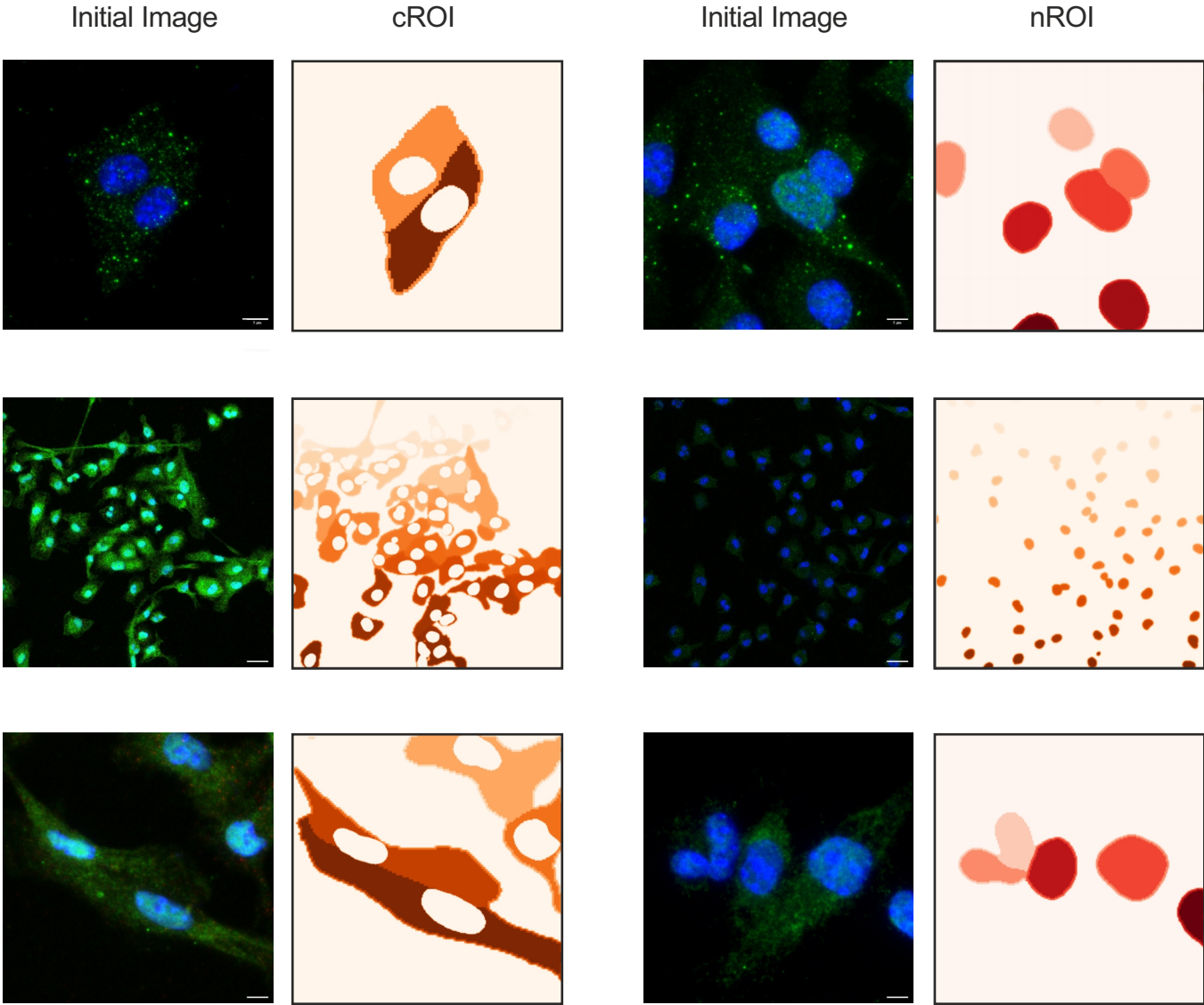

Supplement: Supplementary file 2 — Additional file 2. Graphical User Interface and segmentation. (A) An example of the graphical user interface (GUI). It consists of four separate and interactive panels, which can be used for parameter selection and visual inspection of the analysed images. (B) Segmentation examples of both the cROI and nROI in cells of various sizes. Matplotlib was used for image visualization. [file 12859_2022_4988_MOESM2_ESM.pdf]

A

Green channel

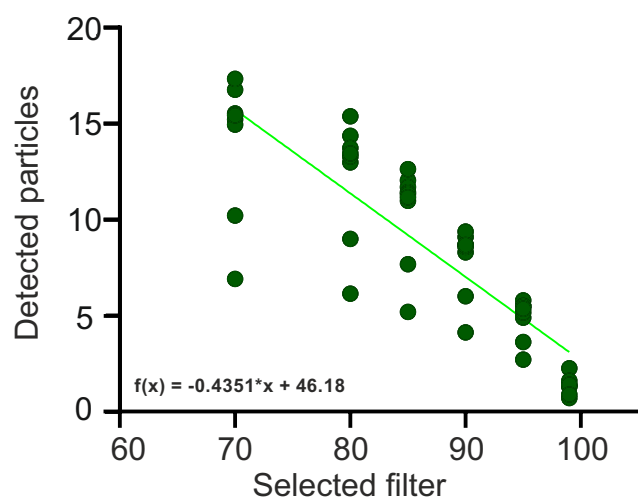

Blue channel

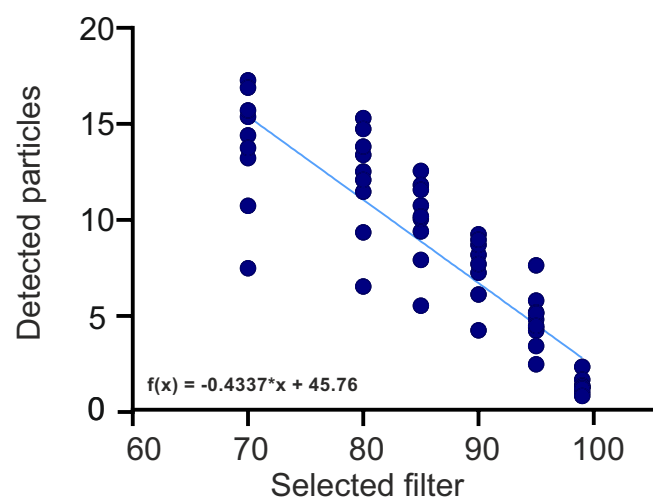

B

Co-localized

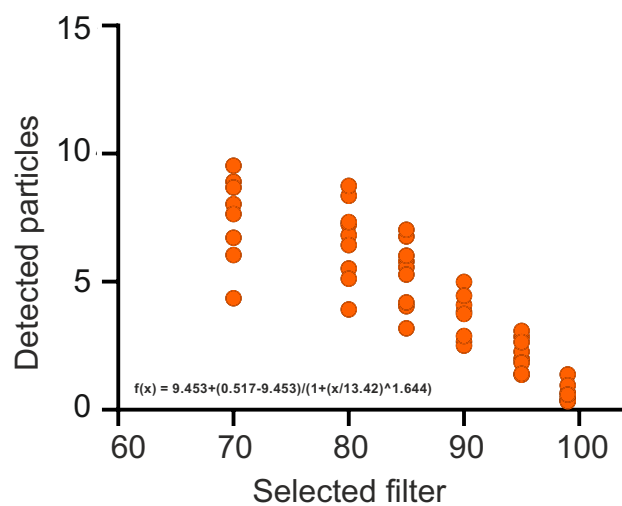

C

Comparison

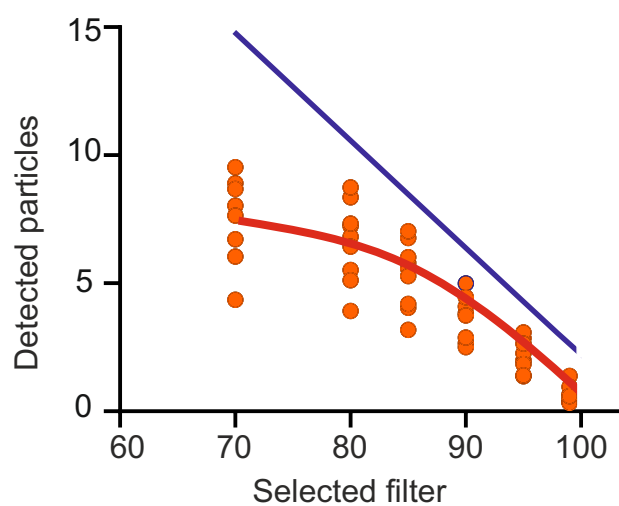

D

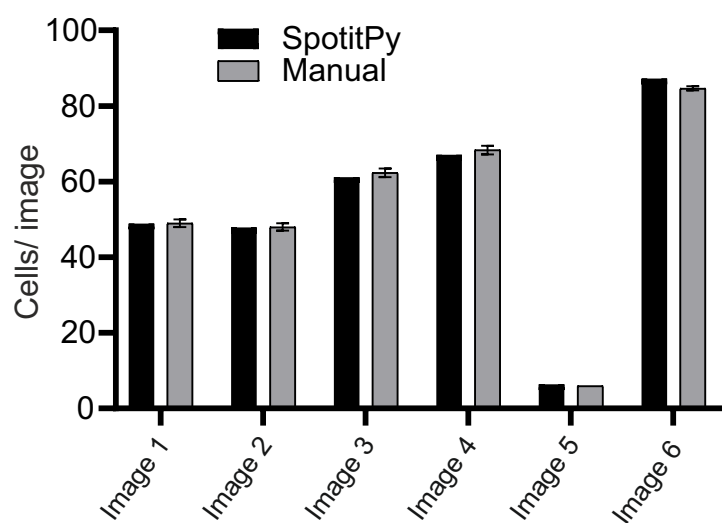

E

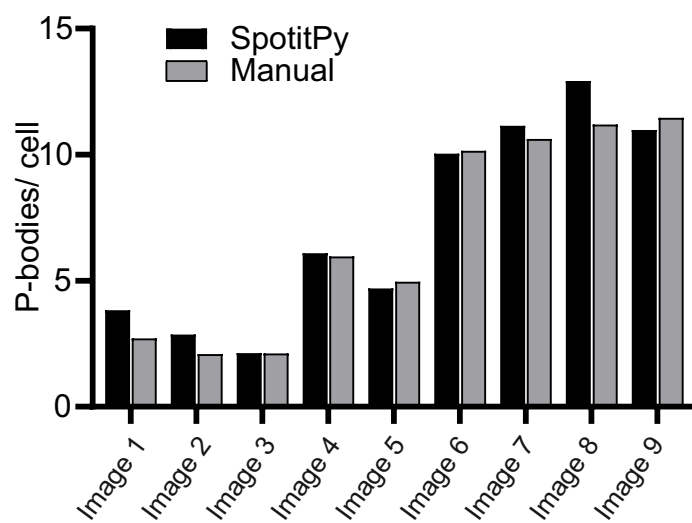

Supplement: Supplementary file 3 — Additional file 3. Enumeration of detected particles and cells. (A) Dot plot depicting the linearity of the percentile selection and particle tracking in both channels. (B) Dot plot depicting the nonlinearity of the percentile selection in the co-localized particles (C) Comparison of the nonlinear trend followed by the identified co-localized particles (red line) and the linear trend of the detected particles in the blue channel (blue line). (D) Comparative analysis of the total number of cells identified in each image by SpotitPy and by manual detection for validation; the dark and grey columns represent the results obtained by SpotitPy and average manual count (n=3), respectively. The images are available in the GitHub repository. (E) Comparative analysis of the total co-localized particle counts of P-bodies in BMDMs by SpotitPy as well as manual count; the dark and grey columns represent the results obtained by SpotitPy and manual count, respectively (n=9). [file 12859_2022_4988_MOESM3_ESM.pdf]
