# Supplementary material for: SpotitPy: a semi-automated tool for object-based co-localization of fluorescent labels in microscopy images
Source: BMC Bioinformatics. 2022 Oct 21;23:439. doi: 10.1186/s12859-022-04988-1 (PMC9587566; doi:10.1186/s12859-022-04988-1)
Supplement: Supplementary file 4 — Additional file 4. Depiction of the software’s output and version’s parameters. (A) Comparison of the graphical user’s interface and command lines interface parameter settings. (B) SpotitPy excel output results. Indices explain each column. [file 12859_2022_4988_MOESM4_ESM.pdf]

A

Parameter	GUI	CLI
Input file	yes	yes
Output file	no	yes
Model	yes	yes
Image selection	yes	yes
Percentile def	yes	yes
Tracking space	no	yes
Gaussian sigma	yes	yes
Channel selection	yes	yes
WBNS noise level	no	yes
Automatic mode	no	yes
Overlap percentage	no	yes

B

Image	Selected nucleus size (px)	Selected particle size (px)	Number of Totally identified cells	Number of positive cells	Cells with 0 signal	Particles per cell in the Blue Channel	Particles per cell in the Green Channel	Particles per positive cells with colocalization	Particles with colocalization per entity of cell
1	60	5	45.1	44.1	1	2.261641	2.350333	1.38322	1.35255
2	60	5	45.61	41.61	4	1.600526	1.688226	0.456621	0.416575
3	60	5	55.14	55.14	0	1.541531	1.396445	0.671019	0.671019
4	60	5	61.1	59.1	2	1.358429	1.309329	0.71066	0.687398
5	200	7	6.32	6.32	0	5.696203	5.379747	3.797468	3.797468
6	60	5	80.38	79.38	1	1.293854	1.318736	0.95742	0.945509
7	60	5	64.9	63.9	1	1.633282	1.186441	0.359937	0.354391
